# Predictors of human immunodeficiency virus (HIV) infection in primary care: a systematic review protocol

**DOI:** 10.1186/s13643-016-0333-2

**Published:** 2016-09-20

**Authors:** Benhildah N. Rumbwere Dube, Tom P. Marshall, Ronan P. Ryan

**Affiliations:** Institute of Applied Health Research, University of Birmingham, Edgbaston, Birmingham, B15 2TT UK

**Keywords:** Acquired immunodeficiency syndrome, Antiretroviral therapies, Diagnosis, Human immunodeficiency virus, Patient characteristics

## Abstract

**Background:**

Antiretroviral therapies for human immunodeficiency virus are more effective if infected individuals are diagnosed early, before they have irreversible immunologic damage. A large proportion of patients that are diagnosed with HIV, in United Kingdom, would have seen a general practitioner (GP) within the previous year. Determining the demographic and clinical characteristics of HIV-infected patients prior to diagnosis of HIV may be useful in identifying patients likely to be HIV positive in primary care. This could help inform a strategy of early HIV testing in primary care. This systematic review aims to identify characteristics of HIV-infected adults prior to diagnosis that could be used in a prediction model for early detection of HIV in primary care.

**Methods:**

The systematic review will search for literature, mainly observational (cohort and case-control) studies, with human participants aged 18 years and over. The exposures are demographic, socio-economic or clinical risk factors or characteristics associated with HIV infection. The comparison group will be patients with no risk factors or no comparison group. The outcome is laboratory-confirmed HIV/AIDS infection. Evidence will be identified from electronic searches of online databases of EMBASE, MEDLINE, The Cochrane Library and grey literature search engines of Open Grey, Web of Science Conference Proceedings Citation Index and examination of reference lists from selected studies (reference searching). Two reviewers will be involved in quality assessment and data extraction of the review. A data extraction form will be developed to collate data from selected studies. A checklist for quality assessment will be adapted from the Scottish Intercollegiate Guidelines Network (SIGN).

**Discussion:**

This systematic review will identify and consolidate existing scientific evidence on characteristics of HIV infected individuals that could be used to inform decision-making in prognostic model development.

**Systematic review registration:**

PROSPERO CRD42016042427

**Electronic supplementary material:**

The online version of this article (doi:10.1186/s13643-016-0333-2) contains supplementary material, which is available to authorized users.

## Background

Human immunodeficiency virus (HIV) is a viral infection that weakens the immune system and is a subsequent causative agent of acquired immunodeficiency syndrome (AIDS) [1]. The virus is transmitted through the exchange of a variety of bodily fluids mainly sexually, perinatal and blood-borne [2, 3]. HIV/AIDS is one of the highest contributor to morbidity, and it is the sixth leading cause of mortality worldwide [2, 4].

The life expectancy of the HIV-infected individuals has increased over the years and is approaching that for the general population [5, 6]. This is a result of the effectiveness of antiretroviral therapies (ARTs) that has led to most individuals coping with HIV infection as a chronic condition rather than an illness inevitably leading to death [7]. The use of ARTs has led to a better quality of life for infected individuals and a reduction in morbidity and mortality [4].

Initially, more focus was placed on HIV prevention strategies and treatment of symptomatic diseases, but due to the benefits of ART, the emphasis has now moved to earlier HIV diagnosis [8]. The CD4 count is an indicator of immunosuppression in an individual infected with HIV [7]. Early diagnosis of people with HIV (CD4 count above 350/mm^3^) improves the effectiveness of antiretroviral therapies, and additionally, the treatment and advice provided reduce onward transmission, thereby making late diagnosis of HIV (CD4 count below 350/mm^3^) an important public health concern [9, 10]. Furthermore, early diagnosis of HIV and earlier use of therapies reduce health and social care costs through prevented illnesses [4, 11]. On the other hand, delayed diagnosis of HIV to late stages (CD4 count below 350/mm^3^) leads to worse prognosis for the patient due to irreversible immunologic damage and associated problems [10, 12].

The methods used in routine HIV testing either involve the use of screening assays on blood for laboratory testing or rapid tests conducted on samples from a finger-prick or mouth swab at point of care. The commonly used and recommended first-line assay tests for HIV antibodies and the HIV p24 antigens simultaneously [7, 13]. These assay tests can be utilised within a month of HIV infection [7, 13]. The sensitivity of these assay tests ranges from 99.8 to 100 %, and the specificity ranges from 99.4 to 100 % [14, 15]. Point-of-care tests (POCTs) are rapid testing devices that diagnose HIV in as short a time as 15 min. However, such tests have lower specificity in comparison to laboratory tests thereby giving significantly high proportion of false positives, especially when used in low prevalence settings [7]. It is therefore possible to test for and diagnose HIV using simple blood tests with few false positives and false negatives.

Public Health England estimated that 107,800 individuals in United Kingdom (UK) were living with HIV in 2013, of which 6000 were newly diagnosed and 24 % were unaware of their HIV status [16]. In 2014, 40 % of people that were newly diagnosed with HIV in UK were detected late (CD4 count below 350/mm^3^), which is an intolerably high proportion [17]. Meanwhile, evidence shows that about 33 % of patients that are diagnosed with HIV in UK would have seen a general practitioner (GP) within the previous year [7, 18, 19]. Therefore, primary care has a role to play in increasing the uptake of HIV diagnostic testing since nearly all UK population is registered with a GP [20]. HIV testing in general practices can be by either sending blood samples for laboratory testing or conducting combined HIV antibody and P24 antigen tests followed by laboratory confirmation [7]. However, among those who visit their GP, a challenge is the fact that HIV/AIDS has many signs and symptoms such as rashes, weight loss and respiratory infections, and these are not specific to HIV/AIDS.

Current UK guidelines recommend HIV testing to individuals from high-risk groups, those with symptoms indicative of HIV or where HIV forms part of the diagnosis [13]. However, 74.2 % of patients who consult their GPs in the period prior to diagnosis do not present these indicator symptoms and diagnoses [19]. This suggests that the predictive factors that are currently in use are not useful in the identification of possible HIV-infected individuals.

A systematic review is therefore necessary to identify demographic, lifestyle, clinical and laboratory characteristics of patients which might be associated with HIV infection in primary care. The identified characteristics, especially from developed countries, will be investigated to determine if they are documented in electronic primary care records and therefore whether information in electronic primary care records can be used to predict which primary care patients have HIV infection. The results of the analysis can identify patients in whom HIV is likely and therefore help inform primary care clinicians which patients they should offer HIV testing in the UK.

This systematic review will identify, critically evaluate and interpret available evidence related to the demographic, lifestyle, clinical and laboratory characteristics associated with HIV/AIDS infection in adults in the developed world [21, 22].

## Methods/design

The steps of this systematic review will include defining the inclusion and exclusion criteria, finding studies, selecting those studies that address the review question and meet the criteria, and extracting and compiling data. This protocol adheres to the requirements of Preferred Reporting Items for Systematic Reviews and Meta-analyses Protocol (PRISMA-P), which is included as an Additional file 1. The review will conform to the requirements of the Preferred Reporting Items for Systematic Reviews and Meta-analyses (PRISMA) [23, 24].

### Research question

This systematic review aims to systematically identify and summarise evidence on characteristics of HIV-infected adults which could be used in the prognostic model for early detection of HIV in primary care. Individual factors in studies with multivariable models of predictors of HIV infection will also be identified.

The review question is:

*What demographic, lifestyle, clinical and laboratory characteristics are associated with HIV infection in adults aged 18 years and over in developed countries?*

### Population, exposure and outcome

This systematic review will consider only studies with human participants aged 18 years and over or those with both adults and children but with results categorised by age groups. The exposures are risk factors or characteristics associated with HIV infection that are demographic, socio-economic or clinical. The comparison group will be patients with no risk factors or no comparison group. The outcome is laboratory-confirmed HIV/AIDS infection. Studies describing frequency, duration and severity of predictive factors will also be included. Thus, the search will cover clinical indicators, socio-economic and demographic, lifestyle-related risk factors and comorbidities and frequency, duration and severity of predictive factors [13] (Additional file 2).

### Study design

The types of studies most suitable for this aetiological review should be mainly observational (analytical) studies that compared groups and produced odds ratios or predictive values or likelihood ratios (case-control and cohort, both retrospective and prospective studies) [25]. However, experimental studies with/without randomisation will also be considered, if the results are in a usable format.

### Search strategy

Studies will be identified and reviewed via electronic searches of online research databases of EMBASE (Ovid), MEDLINE (Ovid) and The Cochrane Library (Wiley). Research bias will be reduced by additional search on unpublished grey literature search engine of Open Grey (SIGLE), Google Scholar and BASE [26, 27]. Additional searches will be conducted on abstracts or conference proceedings using Web of Science Conference Proceedings Citation Index (CPCI), guidelines (NICE, DH) and examination of reference lists from studies included in the review (reference searching) [27]. References will be searched and stored using the Refworks referencing programme hosted by the University of Birmingham.

The review will cover evidence published in any language (English and non-English) so that publication bias is minimised. Although research on signs and symptoms on HIV/AIDS started as early as 1984, changes in the pattern of infection and patient characteristics had occurred ever since, and more evidence was produced afterwards. Hence, the studies covered in this review are those conducted and published from year 1995 onwards.

### Inclusion/exclusion criteria

Only studies undertaken in the following developed countries, Europe (European Union and European Free Trade Association nations) and North America (USA and Canada), Australia and New Zealand, will be included in this review. The main reason for including these developed countries is the difficulties in distinguishing the signs and symptoms of HIV\AIDS in developing countries due to (i) patterns of symptoms and comorbidities, such as TB, in the general population are different in developing and developed countries which will affect the predictive characteristics of those symptoms, (ii) differences in the prevalence of HIV infection which affect the predictive nature of patient characteristics and (iii) patterns of consultation, symptom reporting and laboratory tests and service use are different in countries with good and poor access to primary care affecting the predictive characteristics of these features [2, 3].

Studies which include children only will be excluded. Studies that report on non-HIV diagnosis will also be excluded from this review.

## Data analysis and synthesis

Pre-screening of the searched studies will be the first stage of the analysis where titles/abstracts are assessed to check if they address the review question. A second reviewer will independently check if he/she agrees with the suitability of selected title/abstracts. Differences will be resolved through discussions, and in case of any unresolved disagreements, a third reviewer will be employed.

The next stage is the retrieval of full articles including translation, whenever possible. Another assessment of suitability of study in relation to criteria will be conducted, and the second reviewer will independently check if he/she agrees with the selected studies. Quality assessment of articles will be done with the use of a checklist modified from existing ones such as the Scottish Intercollegiate Guidelines Network (SIGN). This step will ensure that the studies that go through the data extraction process are of good quality and assess risk of bias. Studies will be graded as high quality, acceptable or poor quality. The results from the extraction process will be collated using a data extraction form, to be developed. Description to be documented will include the author, publication year, the study design, number of participants, population under study (country, gender and age) and outcome (including relative risks and models produced). Strengths and weaknesses of the study, including sampling and non-sampling biases should also be documented. Clarity will be requested from the article authors in cases where the information/data is unclear/missing in the study report. Any exclusion of studies at this stage will be documented.

Synthesis of evidence from systematic reviews can either be narrative or statistically analysed (pooling method or meta-analysis), depending on the homogeneity of the studies [28, 29]. Since the results of this systematic review are required to reveal characteristics that can be used in a prognostic model, only tabulation of the results will be produced. The analysis will adhere to the requirements of the PRISMA approach, and a PRISMA diagram will be included, showing the number of studies considered at each level of screening and assessment [24].

## Discussion

The findings from the systematic review will be summarised as tabulations, and the strengths and weaknesses of the review will be discussed. Conclusions and recommendations on existing scientific evidence will be used to inform decision-making in the prediction model development [26]. The patient characteristics will be tabulated according to the following categories

1. Demography and socio-economic (examples: age, gender, ethnicity and deprivation quintiles)
2. Behavioural and lifestyle (examples: numbers and types of sexual partnerships and alcohol and substance misuse)
3. Clinical features, signs and symptoms (examples: weight loss, fever, night sweats and fatigue),
4. Comorbidities (examples: herpes zoster, haemophilia, blood-borne hepatitis, TB, bacterial pneumonia, oral candidiasis and other sexually transmitted infections, frequent diarrhoea consultation and mental disorders).

### Dissemination

The systematic review will be submitted for publication. The findings of the systematic review will contribute to a PhD project and will be presented at conferences.

## Additional files

Additional file 1: PRISMA-P 2015 Checklist. The Preferred Reporting Items for Systematic Reviews and Meta-analyses for Protocols 2015 (PRISMA-P 2015) checklist was used in development of this protocol. Items 2, 4, 5c, 15a-c and 17 were not applicable. (DOCX 29 kb) Additional file 2: Search terms to be used in MEDLINE. The search terms will be modified according to the format of the database search strategy. (DOCX 12 kb)

## Abbreviations

AIDS: Acquired immunodeficiency syndrome
ART: Antiretroviral therapies
GP: General practitioner
HIV: Human immunodeficiency virus
NAAT: Nucleic acid amplification test
PCR: Polymerase chain reaction
POCTs: Point-of-care tests
PRISMA: Preferred Reporting Items for Systematic Reviews and Meta-analyses
PRISMA-P 2015: Preferred Reporting Items for Systematic Reviews and Meta-analyses for Protocols 2015
SIGN: Scottish Intercollegiate Guidelines Network
